# The role of CYP2D in rat brain in methamphetamine-induced striatal dopamine and serotonin release and behavioral sensitization

**DOI:** 10.1007/s00213-021-05808-9

**Published:** 2021-03-01

**Authors:** Marlaina R. Stocco, Ahmed A. El-Sherbeni, Bin Zhao, Maria Novalen, Rachel F. Tyndale

**Affiliations:** 1 Department of Pharmacology and Toxicology, University of Toronto, Toronto, Ontario Canada; 2 Campbell Family Mental Health Research Institute, CAMH, Toronto, Ontario Canada; 3 Department of Clinical Pharmacy, Tanta University, Tanta, Egypt; 4 Department of Psychiatry, University of Toronto, Toronto, Ontario Canada

**Keywords:** CYP2D, Methamphetamine, Behavioral sensitization, Dopamine, Serotonin, Striatum, Metabolism, Neuropharmacology, Microdialysis, Propranolol

## Abstract

**Rationale:**

Cytochrome P450 2D (CYP2D) enzymes metabolize many addictive drugs, including methamphetamine. Variable CYP2D metabolism in the brain may alter CNS drug/metabolite concentrations, consequently affecting addiction liability and neuropsychiatric outcomes; components of these can be modeled by behavioral sensitization in rats.

**Methods:**

To investigate the role of CYP2D in the brain in methamphetamine-induced behavioral sensitization, rats were pretreated centrally with a CYP2D irreversible inhibitor (or vehicle) 20 h prior to each of 7 daily methamphetamine (0.5 mg/kg subcutaneous) injections. In vivo brain microdialysis was used to assess brain drug and metabolite concentrations, and neurotransmitter release.

**Results:**

CYP2D inhibitor (versus vehicle) pretreatment enhanced methamphetamine-induced stereotypy response sensitization. CYP2D inhibitor pretreatment increased brain methamphetamine concentrations and decreased the brain *p*-hydroxylation metabolic ratio. With microdialysis conducted on days 1 and 7, CYP2D inhibitor pretreatment exacerbated stereotypy sensitization and enhanced dopamine and serotonin release in the dorsal striatum. Day 1 brain methamphetamine and amphetamine concentrations correlated with dopamine and serotonin release, which in turn correlated with the stereotypy response slope across sessions (i.e., day 1 through day 7), used as a measure of sensitization.

**Conclusions:**

CYP2D-mediated methamphetamine metabolism in the brain is sufficient to alter behavioral sensitization, brain drug concentrations, and striatal dopamine and serotonin release. Moreover, day 1 methamphetamine-induced neurotransmitter release may be an important predictor of subsequent behavioral sensitization. This suggests the novel contribution of CYP2D in the brain to methamphetamine-induced behavioral sensitization and suggests that the wide variation in human brain CYP2D6 may contribute to differential methamphetamine responses and chronic effects.

**Supplementary Information:**

The online version contains supplementary material available at 10.1007/s00213-021-05808-9.

## Introduction

Methamphetamine (MAMP) is a highly addictive psychostimulant and neurotoxin. In humans, MAMP use can lead to dependence and addiction, characterized by craving and high rates of relapse (Brecht and Herbeck 2014; Lopez et al. 2015). Chronic MAMP use is associated with CNS toxicity, cognitive deficits, and neuropsychiatric conditions, including psychosis (Grant et al. 2012; Henry et al. 2010; London et al. 2015). In animals, repeated MAMP administration induces behavioral sensitization and altered neurotransmission, for example, enhanced dopamine and serotonin release (Ago et al. 2006; Kazahaya et al. 1989). Behavioral sensitization is the progressive augmentation of behavioral response, for example, of stereotypy, following intermittent and repeated drug exposure (Janetsian et al. 2015). Behavioral sensitization has been used to model components of addiction, for example, drug craving and relapse (Robinson and Berridge 1993; Steketee and Kalivas 2011), as well as to model MAMP-induced psychosis and psychotic disorders (Akiyama et al. 1994; Schmidt and Beninger 2006). Identifying factors that influence behavioral sensitization in other species may provide insight into individual differences in susceptibility to chronic effects of MAMP in humans.

MAMP is metabolized by the human cytochrome P450 2D6 (CYP2D6) (Lin et al. 1997). Human CYP2D6 is highly genetically polymorphic, and metabolic phenotypes vary widely (Bertilsson et al. 2002). CYP2D6 is expressed in extrahepatic tissues, such as the brain, where its activity reflects *CYP2D6* genotype but is also influenced by environmental factors, producing greater variation in CYP2D6 in human brains (Chinta et al. 2002; Mann et al. 2008; Miksys and Tyndale 2004). For example, CYP2D6 is higher in the brains of human smokers and human alcoholics (Mann et al. 2008; Miksys et al. 2002), consistent with CYP2D in the brain (but not in the liver) being inducible by nicotine in mice (Singh et al. 2009) and rats (Yue et al. 2008) and by nicotine and alcohol in African Green Monkeys (Miller et al. 2014). The impact of this highly variable CYP2D6 in human brain on MAMP response may be particularly important, because human consumption occurs primarily via routes that bypass first-pass metabolism (Brecht et al. 2004). In rats, CYP2D variation in the brain alters CNS drug concentrations and resulting drug responses (McMillan et al. 2019; Miksys et al. 2017; Zhou et al. 2013), although this has not been examined for amphetamines. By changing brain drug and metabolite concentrations, CYP2D in the brain may influence MAMP-induced neurotransmitter release and behavioral sensitization, depending on the relative contribution of MAMP and its metabolites to these responses.

MAMP is N-demethylated to amphetamine (AMP), and MAMP and AMP are *p*-hydroxylated to *p*-OH-methamphetamine (OH-MAMP) and *p*-OH-amphetamine (OH-AMP), respectively (Lin et al. 1997). These reactions are catalyzed in humans primarily by CYP2D6 and in rats by CYP2D isozymes, with other enzymes contributing to the N-demethylation reaction (Lin et al. 1997; Lin et al. 1995). MAMP and AMP are approximately equipotent in producing behavioral responses and sensitization (Hall et al. 2008). MAMP and AMP induce similar dopamine release in the dorsal striatum, but MAMP induces greater serotonin release in this region (Kuczenski et al. 1995). OH-MAMP and OH-AMP have been implicated in behavioral sensitization due to their potential for accumulation in the brain and their ability to elicit behavioral responses in vivo via dopaminergic and serotonergic mechanisms (Cho et al. 1975; Onogi et al. 2010; Onogi et al. 2011). OH-MAMP and OH-AMP do not readily cross the blood brain barrier due to low lipophilicity, suggesting a potential important contribution of CYP2D within the brain (Kalasz et al. 2009; Kuhn et al. 1978).

Our objectives were to investigate the role of CYP2D in the brain in MAMP-induced acute behavioral response and sensitization and to examine the underlying mechanism by assessing brain drug and metabolite concentrations, as well as dopamine and serotonin release in the dorsal striatum. To assess the role of CYP2D in the brain, intracerebral pretreatment with the CYP2D inhibitor propranolol was administered 20 h prior to MAMP injection to irreversibly inhibit CYP2D in the brain, without affecting CYP2D in the liver and reducing the potential for any effects of propranolol pretreatment, beyond inhibiting CYP2D in the brain, during MAMP administration (McMillan et al. 2019; Miksys et al. 2017). After 20 h, there is negligible propranolol remaining in the brain (~1 h half-life in rat brain), and thus, at the time of MAMP administration, CYP2D in the brain is irreversibly inhibited, and there is likely no remaining propranolol-mediated beta-adrenergic receptor inhibition (Lemmer and Bathe 1982; McMillan et al. 2019; Miksys et al. 2017). We hypothesized that, compared to vehicle pretreatment, CYP2D inhibitor pretreatment would increase MAMP and decrease metabolite concentrations in the brain (but not in serum), enhance striatal dopamine and serotonin release, and enhance MAMP-induced behavioral sensitization. We hypothesized that the increase in brain MAMP concentrations would be associated with enhanced neurotransmitter release and behavioral sensitization, due to a greater contribution of MAMP (versus its metabolites) to these responses.

## Methods

### Animals

Adult male Wistar rats (Charles River, Saint-Constant, QC, Canada) were housed in groups of 1–3 and kept under a 12-h light/dark cycle with testing during the light phase. Water was provided ad libitum, and food was restricted to limit weight gain throughout experiments. All procedures were approved by the Animal Care Committee at the University of Toronto, and were conducted in accordance with the guidelines of the Canadian Council on Animal Care.

### Cannulation surgery

Rats were anesthetized with isoflurane and implanted with stainless steel guide cannulas into the lateral ventricle (anterior-posterior −0.9 mm, lateral +1.4 mm, dorsoventral −3.6 mm) and/or the right dorsal striatum (anterior-posterior +1.2 mm, lateral −3.0 mm, dorsoventral −3.6 mm) for intracerebral injections (P1 Technologies, Roanoke, VA, USA) or for the insertion of microdialysis probes (MD-2257, BASi, West Lafayette, IN, USA). In experiment 1, rats were cannulated into the lateral ventricle for intracerebral injections. In experiment 2, rats were cannulated into the lateral ventricle or the dorsal striatum for intracerebral injections and insertion of microdialysis probes. In experiment 3, rats were cannulated into the lateral ventricle for intracerebral injections and into the dorsal striatum for insertion of microdialysis probes. Small stabilization screws and dental cement were used to secure guide cannulas to the skull, as previously described (McMillan and Tyndale 2015). Animals recovered for 7 days prior to initiating experimental procedures.

### Drug treatment

Propranolol hydrochloride (Sigma, Oakville, ON, Canada) was dissolved in a 20% (w/v) solution of 2-hydroxypropyl-β-cyclodextrin (Sigma, Oakville, ON, Canada) in distilled water, to a final concentration of 5 μg propranolol base/μl cyclodextrin vehicle. A volume of 4 μl (i.e., 20 μg propranolol total) was injected intracerebroventricularly (ICV) or intrastriatally (IST) via guide cannula 20 h prior to MAMP injection. Propranolol undergoes metabolism by CYP2D, producing a reactive intermediate that binds covalently to the active site, yielding irreversible inhibition of CYP2D that remains (1) after propranolol no longer remains in the brain (~1 h half-life in rat brain) and (2) until there is new CYP2D protein turnover (Lemmer and Bathe 1982; Masubuchi et al. 1994). MAMP hydrochloride (Sigma, St. Louis, MO, USA) was dissolved in saline (0.9% NaCl, pH 7) and injected subcutaneously (SC) at a dose 0.5 mg base/kg body weight.

### Microdialysis procedures and analysis

Microdialysis probes (concentric silica-coated, 2 mm membrane; MD-2201, BASi) were inserted via the guide cannula, and Ringer’s solution (147 mM Na^+^, 2 mM Ca^2+^, 4 mM K^+^, 155 mM Cl^-^, 0.1 mM ascorbic acid) was perfused at a constant flow rate of 2 μl/min. Dialysate was collected on ice before and after MAMP injection to assess brain concentrations of MAMP and its metabolites, AMP and OH-MAMP, as well as dopamine and serotonin. As previously described (El-Sherbeni et al. 2020), a solution containing internal standards (1 ng/ml final concentration of methamphetamine-D5, amphetamine-D6, dopamine-D4, and serotonin-D4), EDTA (90 μg/ml final concentration), ascorbic acid (81 μg/ml final concentration), and acetic acid (0.9% final concentration) was added to the dialysate samples (1:9 v/v). Samples were then analyzed using liquid chromatography-electrospray ionization-tandem mass spectrometry (Agilent 1260 LC system coupled with Agilent 6430 Triple Quadrupole system, Agilent Technologies, Santa Clara, CA, USA). The limit of quantitation (LOQ) was 0.005 ng/ml for all analytes. The concentrations of MAMP, AMP, and OH-MAMP were corrected for 14.3%, 13.1%, and 19.5% probe recovery, respectively, assessed in vitro, as previously described (El-Sherbeni et al. 2020). Dopamine and serotonin data were presented as the percentage of baseline rather than as concentrations, so corrections were not needed.

### Serum drug concentrations

Saphenous vein blood samples were collected 100 and 130 min after MAMP injection to assess circulating concentrations of MAMP and its metabolites. Blood samples were centrifuged at 5000*g* for 10 min, and serum was collected. As previously described (Hendrickson et al. 2006), a solution containing internal standards (20 ng/ml final concentration of methamphetamine-D5 and amphetamine D-6) was added to serum samples (1:9 v/v). Serum protein was precipitated by the addition of equal volumes of 20% trichloroacetic acid in water and centrifugation at 9500*g* for 15 min. The supernatants were collected and analyzed by LCMS (Agilent Technologies), using a BDS Hypersil C8 column to achieve chromatographic separation (3 μm, 100 × 2.1 mm, Thermo Scientific, College Park, GA, USA). Gas temperature, flow, and pressure were set at 350°C, 10 l/min, and 30 psi, respectively, and capillary voltage was 3.0 kV. The LOQ of all analytes was 1 ng/ml.

### Experiment 1: behavior

Propranolol (20 μg; *n* = 11) or vehicle (*n* = 12) pretreatment was administered ICV 20 h prior to MAMP (0.5 mg/kg SC) injections, which were administered once daily for 7 days. For feasibility, the experiment was performed in two sets (*n* = 5–6 animals per pretreatment per set), and the data were combined. During each MAMP session, stereotypy time and rearing events were assessed in 5-min bins for 30 min prior to, and 120 min after, MAMP injection using an infrared beam open field system (Superflex Open-Field and Sensors, Fusion v4 Software; Omnitech Electronics Inc., Columbus, OH, USA). The Omnitech system defined stereotypy as repeated breaking of one beam or a set of beams only on the horizontal plane (basal sensors) and defined rearing as any breaking of beams on the vertical plane (elevated sensors).

### Experiment 2: microdialysis

Propranolol (20 μg) or vehicle pretreatment was administered ICV or IST 20 h prior to MAMP (0.5 mg/kg SC), such that pretreatment and delivery site were balanced (i.e., ICV propranolol, ICV vehicle, IST propranolol, IST vehicle, all *n*=3). Dialysate was collected ICV or IST (i.e., same as the pretreatment delivery site) in 15-min bins for 75 min prior to, and 120 min after, MAMP injection, and blood samples were taken at 100 and 130 min after MAMP injection.

### Experiment 3: behavior with microdialysis

Propranolol (20 μg; *n* = 8) or vehicle (*n* = 8) pretreatment was administered ICV 20 h prior to MAMP (0.5 mg/kg SC) injections, which were administered once daily for 7 days. For feasibility, the experiment was performed in two sets (*n* = 4 animals per pretreatment per set), and the data were combined. Following MAMP injection, stereotypy time was assessed from 30 to 50 min, and rearing events from 15 to 45 min, to capture initial peak responses (based on behavior in experiment 1). Stereotypy and rearing responses were scored from video recordings taken during each MAMP session to allow for behavior recording and microdialysis to occur simultaneously on days 1 and 7 and for a consistent environment to be maintained from days 1 through 7. Stereotypy was defined as performance of repetitive and focused behaviors, including head bobbing or swinging, sniffing, grooming, and masticating, whether in the horizontal plane or while rearing. A rearing event was defined as both front paws lifting from the floor and being replaced, independent of other behaviors (i.e., not if occurring while ambulating or grooming). A subset of videos was scored by a second blinded observer, and in the small proportion of cases where scores differed by more than 10 s of stereotypy time or more than 5 rearing events, the average of both scores was used.

#### Microdialysis

On days 1 and 7, dialysate was collected IST in 15-min bins for 45 min prior to, and 90 min after, MAMP injection, and blood samples were taken at 100 and 130 min after MAMP injection. Dopamine and serotonin release were calculated as the percentage of baseline (%baseline) concentration values. For dopamine, the 3 baseline (i.e., pre-MAMP injection) samples were averaged. For serotonin, only the last baseline sample was used, due to high serotonin concentrations in the first two baseline samples. All samples collected post-MAMP from each animal were represented as a percentage of its baseline value for subsequent analyses and graphing. Microdialysis data for all analytes were removed in three instances, either due to the formation of a cyst at the base of the microdialysis guide cannula (*n* = 1 animal’s data on day 7 only) or due to the presence of blood in the cannula during microdialysis resulting in unreliable analyte concentrations, i.e., serotonin >20 times higher than other samples (*n* = 1 animal’s data on day 1 only; *n* = 1 animal’s data on day 7 only).

### Statistical analysis

Data were analyzed using Prism6 software (GraphPad version 6.0c, La Jolla, CA, USA) by mixed ANOVA, two-way ANOVA, or unpaired two-tailed *t* tests, where appropriate. Relationships between data were assessed using Pearson correlation coefficients. Stereotypy and rearing responses (experiments 1 and 3) were analyzed using mixed ANOVAs. Bonferroni post hoc tests were used to make pairwise comparisons between pretreatments, and Dunnett’s post hoc tests were used to compare behavioral responses between sessions within pretreatment. Slopes (stereotypy time (s)/day or rears/day) were compared between pretreatments using unpaired two-tailed *t*-tests with Welch’s correction applied if the *F*-test comparing variances was significant. Slopes represent that of the linear regression line through the given data points. Analytes in brain dialysate or in serum (experiments 2 and 3) and metabolic ratios (i.e., AMP/MAMP and OH-MAMP/MAMP) were compared between pretreatments across time using two-way or mixed ANOVAs, as indicated in the results. Serum MAMP slopes (ng/ml/min) were compared between pretreatments using unpaired two-tailed *t*-tests with Welch’s correction applied when appropriate. Dopamine and serotonin release (assessed as %baseline) were compared between pretreatments across time using mixed ANOVAs, and area under the curve (AUC) was compared between pretreatments and between sessions using two-way ANOVAs with Bonferroni post hoc tests. AUC values were calculated using the linear trapezoid rule. For brain analytes in experiment 3, AUC values were normalized between the two experiment sets (i.e., values from the first set were normalized to the vehicle group of the second set) to account for inter-day variation in LCMS analysis, due to the dialysate samples from the two sets being analyzed separately.

## Results

### Experiment 1: propranolol pretreatment enhanced methamphetamine-induced stereotypy and rearing

**Fig. 1:**
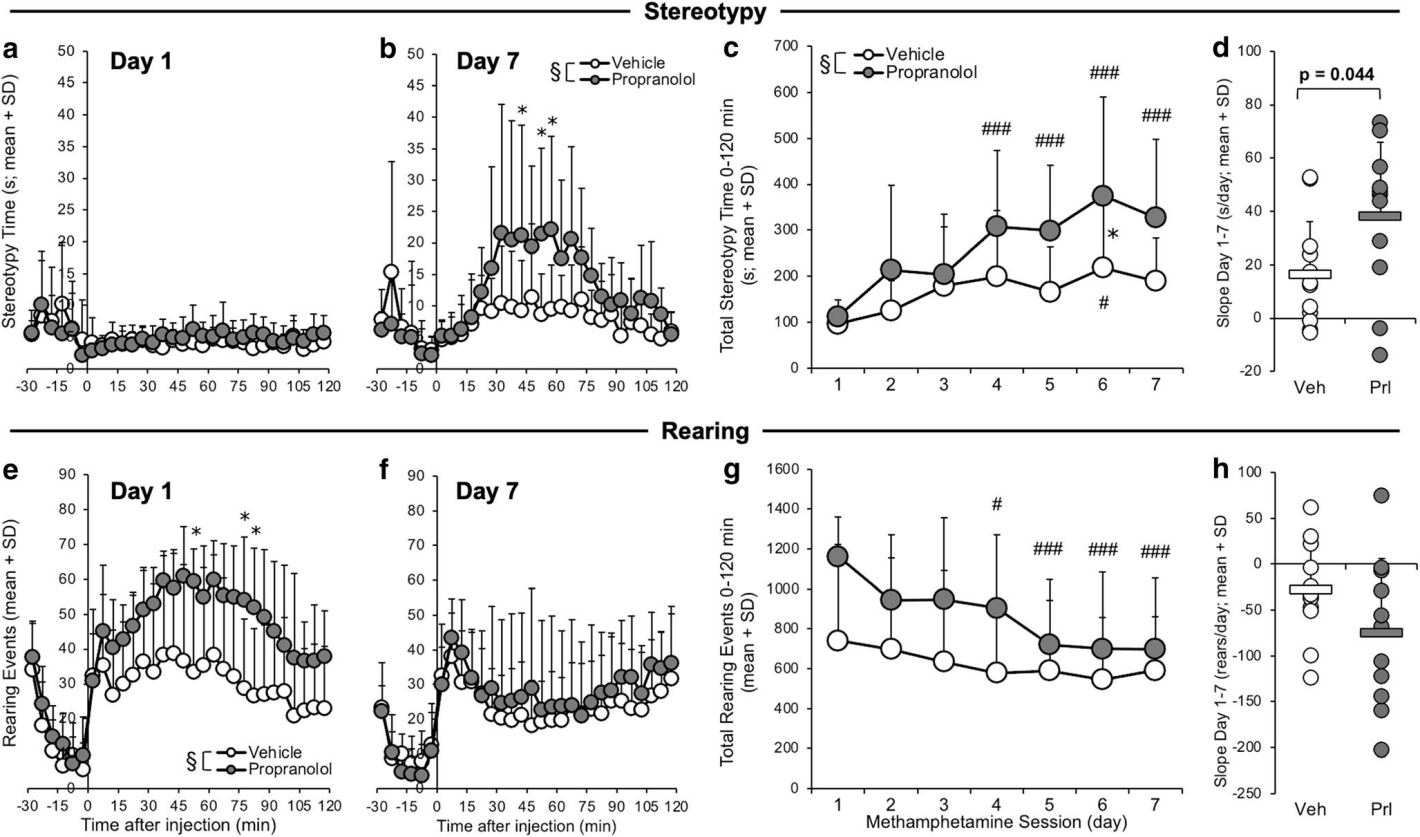
ICV propranolol (versus vehicle) pretreatment enhanced MAMP-induced stereotypy and rearing responses. Rats were given ICV propranolol (*n* = 11) or vehicle (*n* = 12) pretreatment 20 h prior to 7 daily MAMP injections, and stereotypy and rearing responses were recorded daily for 120 min (experiment 1). Stereotypy time on (**a**) day 1 did not differ between pretreatments and on (**b**) day 7 was higher in propranolol-pretreated rats. (**c**) Total stereotypy time increased across MAMP sessions and was higher propranolol-pretreated rats. (**d**) The slope of stereotypy time across sessions was higher in propranolol-pretreated rats. Rearing events on (**e**) day 1 were higher in propranolol-pretreated rats and on (**f**) day 7 did not differ between pretreatments. (**g**) Total rearing events decreased across MAMP sessions only in propranolol-pretreated rats. (**h**) The slope of rearing events across sessions did not differ between pretreatments. Main effect of pretreatment: §*p* < 0.05. Propranolol versus vehicle: *p < 0.05. Day versus day 1: #*p* < 0.05, ###*p* < 0.001. Veh, vehicle; Prl, propranolol; SD, standard deviation

The impact of ICV propranolol compared to vehicle pretreatment on daily MAMP-induced stereotypy and rearing responses was assessed. MAMP-induced stereotypy time did not differ between pretreatments on day 1 (Fig. 1a), but it was higher in rats given propranolol pretreatment on day 7 (pretreatment, *F*_(1,21)_ = 5.85, *p* = 0.025) (Fig. 1b). Total stereotypy time increased across daily MAMP sessions (session, *F*_(6,126)_ = 9.97, *p* < 0.001) to a greater extent in propranolol-pretreated rats (day 1 versus days 4, 5, 6, and 7, *p* < 0.001) compared to vehicle-pretreated rats (day 1 versus day 6, *p* = 0.015) (Fig. 1c). Compared to vehicle pretreatment, propranolol pretreatment enhanced total stereotypy time across sessions (pretreatment, *F*_(1,21)_ = 5.30, *p* = 0.032). The slope of stereotypy time across sessions was also higher in rats given propranolol pretreatment (*t*_(21)_=2.15, *p*=0.044) (Fig. 1d). This suggests that stereotypy response sensitized across daily MAMP sessions and was enhanced by propranolol pretreatment.

MAMP-induced rearing events were higher in rats given ICV propranolol pretreatment on day 1 (pretreatment, *F*_(1,21)_ = 7.23, *p* = 0.014) (Fig. 1e), but did not differ between pretreatments on day 7 (Fig. 1f). Total rearing events decreased across daily MAMP sessions (session, *F*_(6,126)_ = 6.30, *p* < 0.001) only in propranolol-pretreated rats (day 1 versus day 4, *p* = 0.041; days 5, 6, and 7, *p* < 0.001) (Fig. 1g). Compared to vehicle pretreatment, propranolol pretreatment trended towards enhancing total rearing events across sessions (pretreatment, *F*_(1,21)_ = 3.61, *p* = 0.071). The slope of rearing events across sessions did not differ between pretreatments (Fig. 1h). This suggests that rearing response was initially enhanced by propranolol pretreatment and then decreased across daily MAMP sessions in rats given propranolol pretreatment.

There was no effect of ICV propranolol pretreatment on baseline (i.e., pre-MAMP injection) stereotypy and rearing responses (pretreatment, all *p* > 0.05). This suggests that propranolol pretreatment, administered 20 h prior to MAMP injection and cleared from the brain due to its short half-life in rats (~1 h) (Bianchetti et al. 1980; Lemmer and Bathe 1982), had no relevant effects, beyond inhibiting CYP2D in brain, on these behaviors.

### Experiment 2: propranolol pretreatment increased brain methamphetamine concentrations

**Fig. 2:**
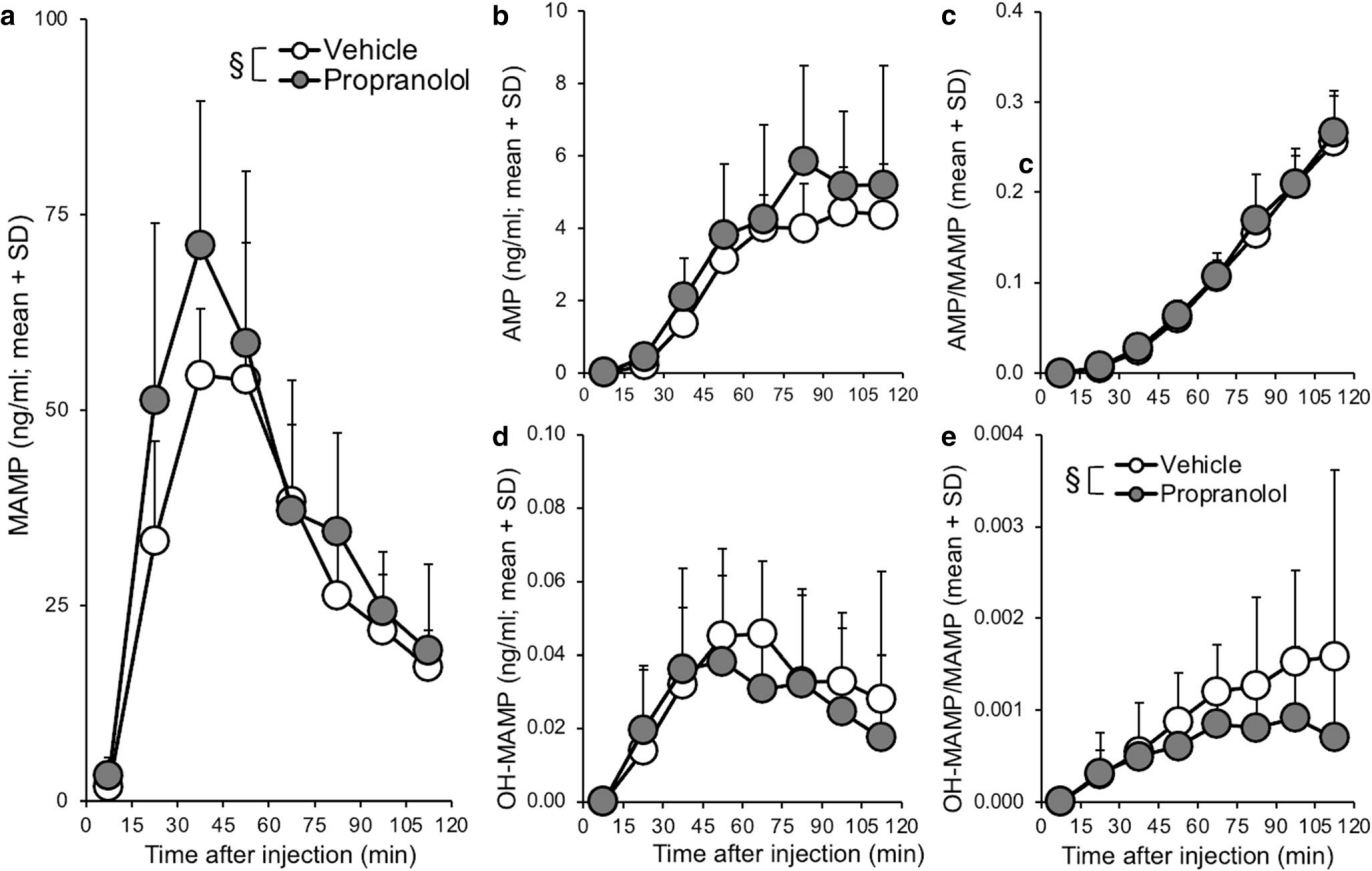
ICV and IST propranolol (versus vehicle) pretreatment increased brain MAMP and decreased the brain OH-MAMP/MAMP ratio. Rats were given propranolol (*n* = 3 ICV, 3 IST) or vehicle (*n* = 3 ICV, 3 IST) pretreatment 20 h prior to a single MAMP injection (experiment 2). (**a**) MAMP concentrations in brain dialysate were higher in propranolol-pretreated rats. While (**b**) AMP, (**c**) the AMP/MAMP ratio, and (**d**) OH-MAMP in brain dialysate did not differ between pretreatments, (**e**) the OH-MAMP/MAMP ratio was lower in propranolol-pretreated rats. One sample (0–15 min) from *n*=1 animal was not collected, and no analytes could be reported for the sample; OH-MAMP in three samples (75–120 min) from *n*=1 animal were omitted due to issues with collection yielding unreliable analyte concentrations (i.e., ~50 times higher than other samples). Main effect of pretreatment: §*p* < 0.05. SD, standard deviation

The impact of propranolol compared to vehicle pretreatment on brain MAMP and metabolite concentrations was assessed. There were no differences within pretreatment among analytes collected ICV versus IST (region, all *p* > 0.05), so the data were combined for analysis of pretreatment effects. Propranolol pretreatment increased MAMP concentrations in brain dialysate (two-way ANOVA; pretreatment, *F*_(1,79)_ = 5.78, *p* = 0.019) (Fig. 2a). There was no effect of propranolol pretreatment on brain concentrations of AMP (Fig. 2b) or on the AMP/MAMP metabolic ratio (Fig. 2c), a measure of in vivo CYP2D-mediated MAMP *N*-demethylation. There was no effect of propranolol pretreatment on brain concentrations of OH-MAMP (Fig. 2d), but the OH-MAMP/MAMP metabolic ratio, a measure of in vivo CYP2D-mediated MAMP *p*-hydroxylation, was lower in rats given propranolol pretreatment (two-way ANOVA; pretreatment, *F*_(1,76)_ = 4.32, *p* = 0.041) (Fig. 2e). This suggests that propranolol pretreatment increased brain MAMP concentrations and decreased CYP2D-mediated MAMP *p*-hydroxylation to OH-MAMP.

Blood samples were collected to verify that there was no impact of propranolol pretreatment on peripheral drug concentrations. There was no effect of pretreatment on serum MAMP (Online Resource 1a), serum AMP (Online Resource 1b), or the AMP/MAMP ratio (Online Resource 1c) (pretreatment, all *p* > 0.05). There was also no difference between the slope of MAMP disappearance in rats given propranolol (−0.42 ± 0.61) versus vehicle (−0.49 ± 0.52) pretreatment (data not shown). This suggests that following ICV or IST propranolol pretreatment, propranolol did not cross into the peripheral system in sufficient amounts to inhibit CYP2D in the liver and alter circulating drug and metabolite concentrations.

### Experiment 3A: propranolol pretreatment enhanced methamphetamine-induced stereotypy sensitization

**Fig. 3:**
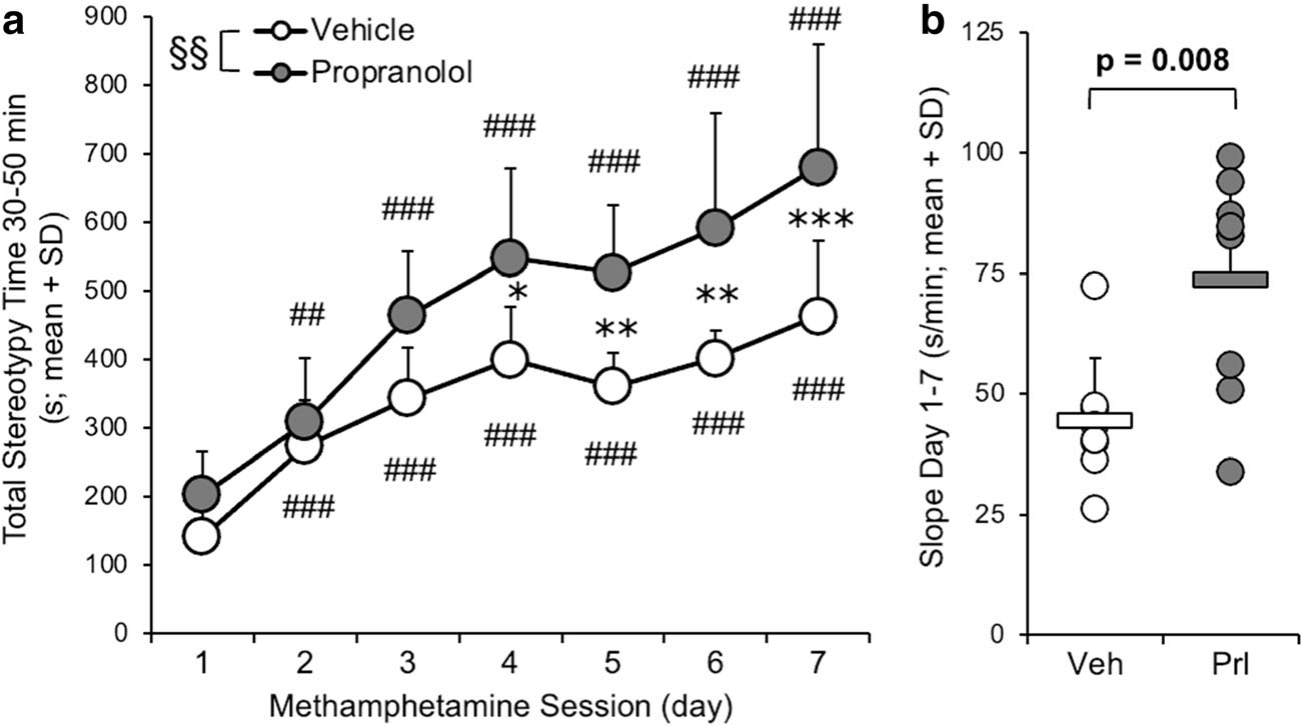
ICV propranolol (versus vehicle) pretreatment enhanced MAMP-induced stereotypy response. Rats were given ICV propranolol (*n* = 8) or vehicle (*n* = 8) pretreatment 20 h prior to 7 daily MAMP injections, and stereotypy response was recorded daily from 30 to 50 min after injection (experiment 3). **a** Total stereotypy time increased across MAMP sessions and was higher in propranolol-pretreated rats. **b** The slope of stereotypy time across sessions was higher in propranolol-pretreated rats. Main effect of pretreatment: §§*p* < 0.01. Propranolol versus vehicle: *p < 0.05, **p < 0.01, ***p < 0.001. Day versus day 1: ##*p* < 0.01, ###*p* < 0.001. Veh, vehicle; Prl, propranolol; SD, standard deviation

The impact of ICV propranolol compared to vehicle pretreatment on daily MAMP-induced behavioral responses was assessed, and IST microdialysis was conducted on days 1 and 7. Total stereotypy time, assessed 30–50 min after MAMP injection, increased across daily MAMP sessions (session, *F*_(6,84)_ = 77.7, *p* < 0.001) in propranolol-pretreated rats (day 1 versus day 2, *p* = 0.005; days 3, 4, 5, 6, and 7, all *p* < 0.001) and vehicle-pretreated rats (day 1 versus days 2, 3, 4, 5, 6, and 7, *p* < 0.001) (Fig. 3a). Compared to vehicle pretreatment, propranolol pretreatment enhanced total stereotypy time across sessions (pretreatment, *F*_(1,14)_ = 10.2, *p* = 0.006). The slope of stereotypy time across sessions was also higher in rats given propranolol pretreatment (*t*_(14)_ = 3.07, *p* = 0.008) (Fig. 3b). Thus, in the presence of microdialysis on days 1 and 7, stereotypy response sensitized across daily MAMP sessions and was enhanced by propranolol pretreatment, consistent with the data when behavior was assessed in the absence of microdialysis (experiment 1, Fig. 1). Likewise, in the presence of microdialysis on days 1 and 7, rearing events decreased across daily MAMP sessions (session, F_(5,70)_ = 4.81, *p* = 0.001) only in propranolol-pretreated rats (day 2 versus day 4, *p* = 0.045; day 5, *p* = 0.006; day 6, *p* = 0.003; day 7, *p* < 0.001) (Online Resource 2), similar to when rearing was assessed in the absence of microdialysis (experiment 1, Fig. 1).

### Experiment 3B: propranolol pretreatment enhanced methamphetamine-induced dopamine and serotonin release

**Fig. 4:**
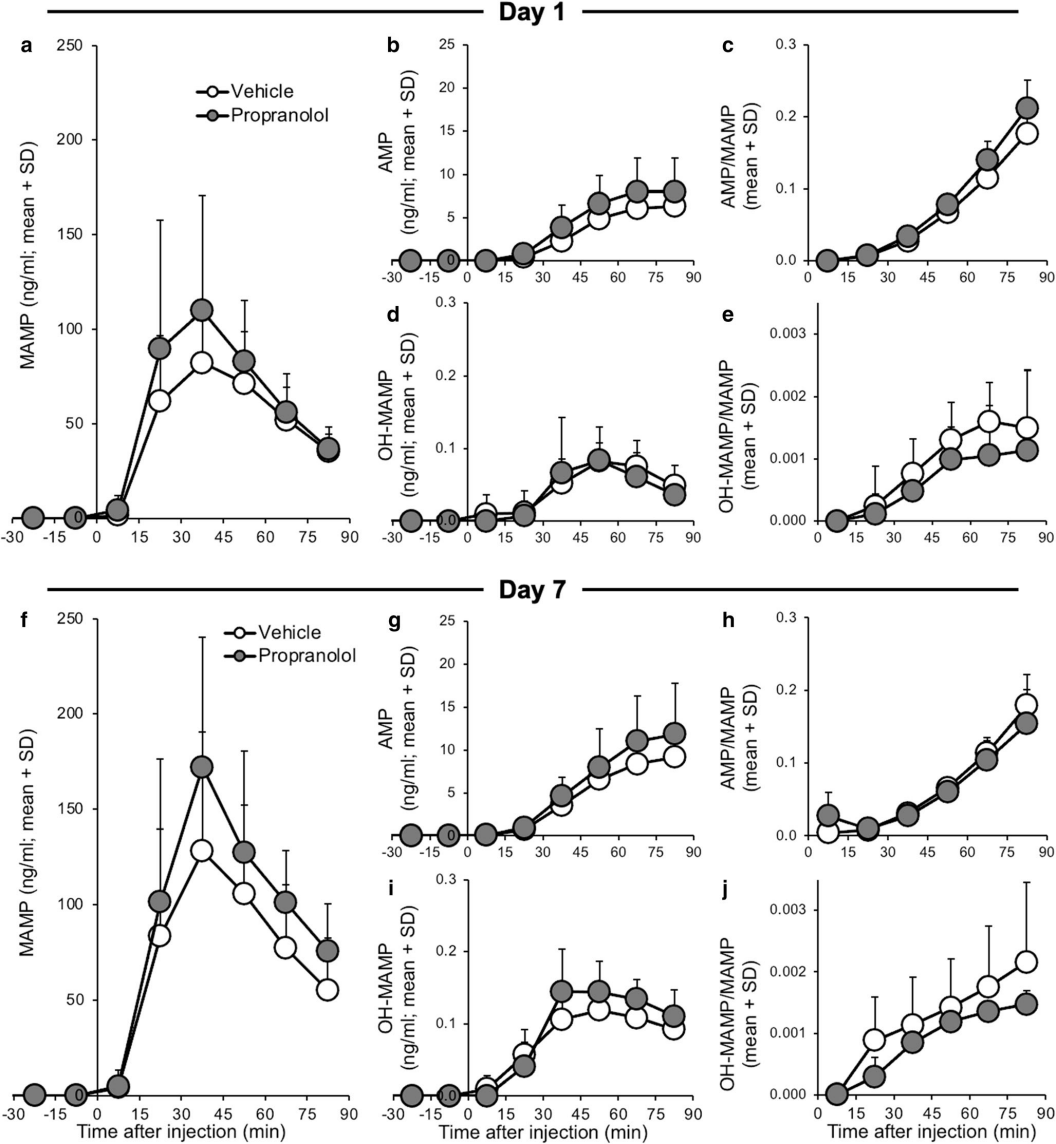
ICV propranolol (versus vehicle) pretreatment did not significantly alter brain MAMP and metabolite concentrations. Rats were given ICV propranolol or vehicle pretreatment 20 h prior to 7 daily MAMP sessions, and IST microdialysis was conducted on days 1 (top) and 7 (bottom) (experiment 3). **a, f** MAMP, **b, g** AMP, **c, h** the AMP/MAMP ratio, **d, i** OH-MAMP, and **e, j** the OH-MAMP/MAMP ratio in brain dialysate did not differ significantly between pretreatments. Day 1: *n* = 8 propranolol, 7 vehicle. Day 7: *n* = 6 propranolol, 8 vehicle. SD, standard deviation

**Fig. 5:**
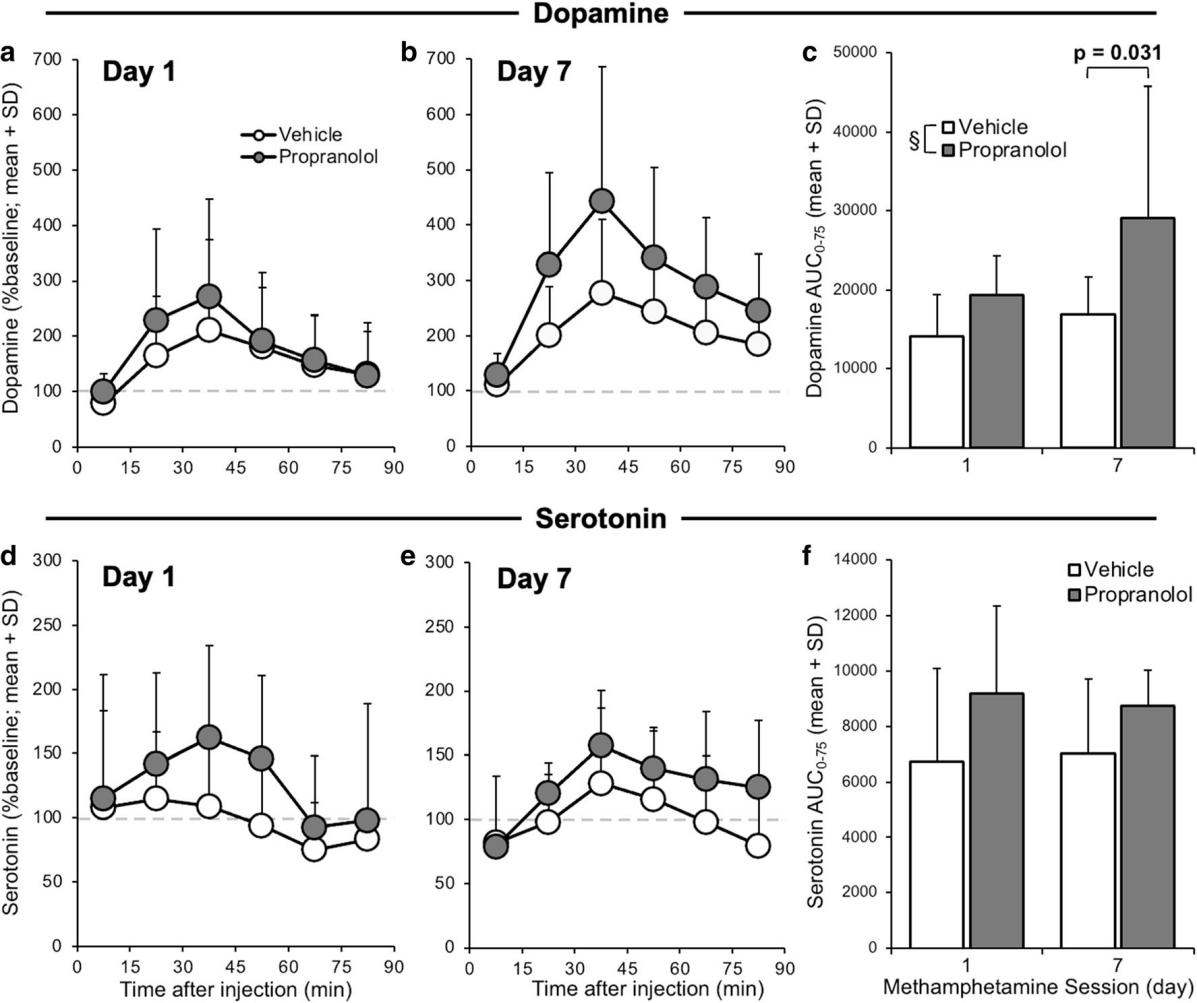
ICV propranolol (versus vehicle) pretreatment enhanced MAMP-induced dopamine and serotonin release. Rats were given ICV propranolol or vehicle pretreatment 20 h prior to 7 daily MAMP sessions, and IST microdialysis was conducted on days 1 and 7 to measure dopamine (top) and serotonin (bottom) (experiment 3). Dopamine release (%baseline) did not differ between pretreatments on (**a**) day 1 or (**b**) day 7. (**c**) Dopamine AUC_0-75_ (area under the %baseline-time curve) was higher in propranolol-pretreated rats and trended towards increasing between sessions. Serotonin release (%baseline) did not differ between pretreatments on (**d**) day 1 or (**e**) day 7. (**f**) Serotonin AUC_0-75_ (area under the %baseline-time curve) trended towards being higher in propranolol-pretreated rats. Day 1: *n* = 8 propranolol, 7 vehicle. Day 7: *n* = 6 propranolol, 8 vehicle. Main effect of pretreatment: §*p* < 0.05. SD, standard deviation

While the impact of ICV propranolol compared to vehicle pretreatment on brain MAMP did not reach significance (nor on brain metabolites and the brain metabolic ratios) (mixed ANOVAs; pretreatment, all *p* > 0.05) on day 1 or day 7 (Fig. 4), there was an impact of propranolol pretreatment on striatal dopamine and serotonin release (Fig. 5). There was a significant effect of pretreatment on dopamine AUC_0-75_ (*F*_(1,25)_ = 7.20, *p* = 0.013), due to ICV propranolol pretreatment increasing AUC_0-75_ (day 7, *p* = 0.031). Dopamine AUC_0-75_ also trended towards increasing between days 1 and 7 (session, *F*_(1,25)_ = 3.65, *p* = 0.068) (Fig. 5c). Propranolol pretreatment trended towards increasing serotonin AUC_0-75_ (pretreatment, *F*_(1,25)_ = 3.93, *p* = 0.059) (Fig. 5f). This suggests that propranolol pretreatment enhanced dopamine and serotonin release and that dopamine release sensitized following daily MAMP sessions.

There was no effect of ICV pretreatment on baseline (i.e., pre-MAMP injection) dopamine or serotonin concentrations on day 1 or day 7 (pretreatment, all *p* > 0.05). This suggests that ICV propranolol pretreatment had no relevant effects, beyond inhibiting CYP2D in brain, on basal concentrations of these neurotransmitters in the dorsal striatum. As expected, there was no effect of ICV propranolol pretreatment on serum drug concentrations (pretreatment, all *p* > 0.05) on day 1 or on day 7 (Online Resource 3).

### Experiment 3C: day 1 brain MAMP- and AMP-induced dopamine and serotonin release were associated with stereotypy sensitization

**Fig. 6:**
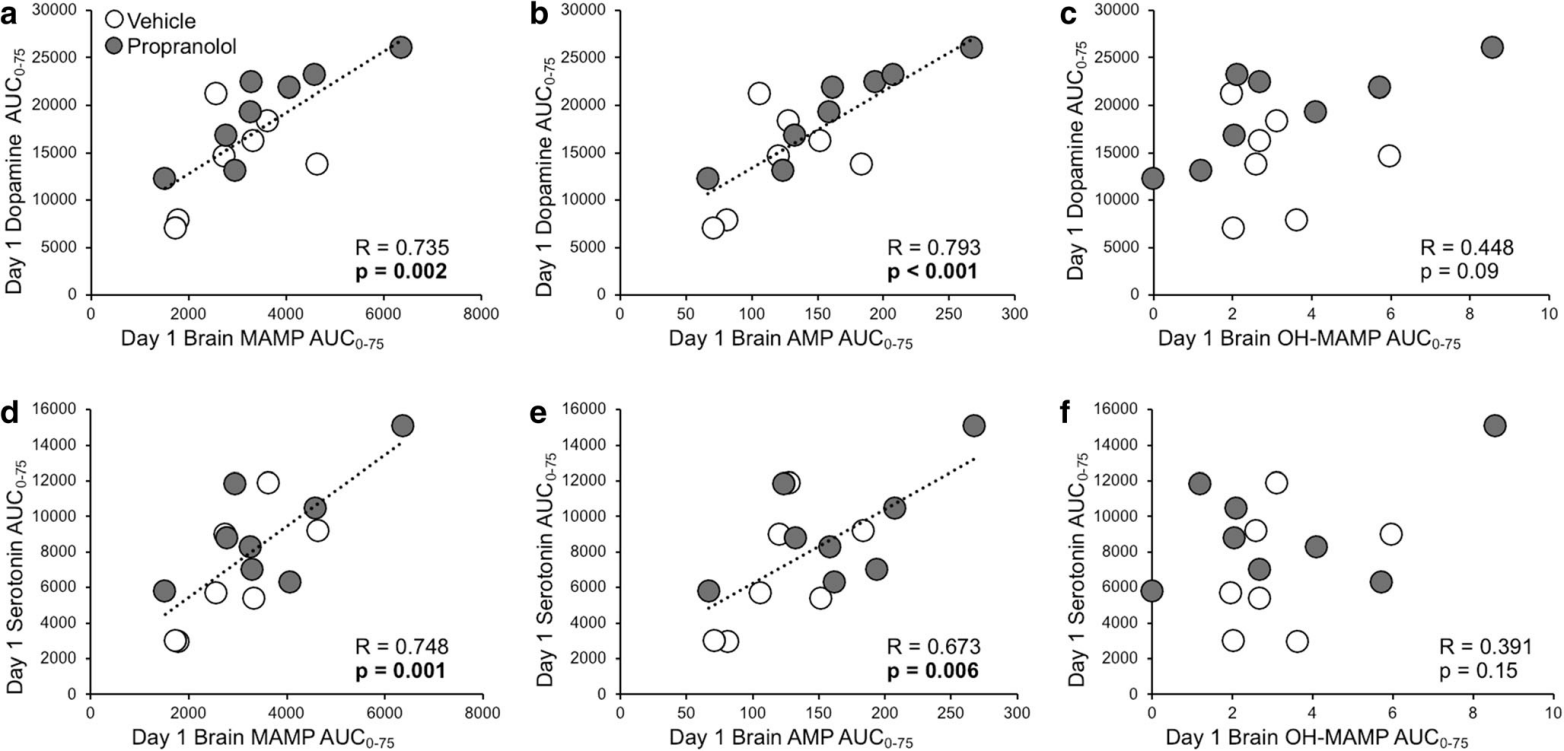
Day 1 dopamine and serotonin correlated with brain MAMP and AMP. Rats were given ICV propranolol or vehicle pretreatment 20 h prior to 7 daily MAMP sessions, and IST microdialysis was conducted on days 1 and 7 (experiment 3). Day 1 dopamine AUC_0-75_ correlated with day 1 brain (**a**) MAMP and (**b**) AMP, but not with (**c**) OH-MAMP AUC_0-75_. Day 1 serotonin AUC_0-75_ correlated with day 1 brain (**d**) MAMP and (**e**) AMP, but not with (**f**) OH-MAMP AUC_0-75_. Correlations were assessed with pretreatment groups combined (*n* = 8 propranolol, 7 vehicle). SD, standard deviation

To investigate whether MAMP or its metabolites were responsible for neurotransmitter release, relationships between AUC_0-75_ were assessed for brain MAMP, AMP, and OH-MAMP, with day 1 dopamine and serotonin release (Fig. 6). AUC_0-75_ was used because it captured peak MAMP concentrations, as well as peak dopamine and serotonin responses. Day 1 dopamine release correlated with brain MAMP (*R* = 0.735, *p* = 0.002) and AMP (*R* = 0.793, *p* < 0.001) and trended towards correlating with OH-MAMP (*R* = 0.448, *p* = 0.094) (Fig. 6a–c). Day 1 serotonin release correlated with brain MAMP (*R* = 0.748, *p* = 0.001) and AMP (*R* = 0.673, *p* = 0.006) but did not correlate with OH-MAMP (*R* = 0.391, *p* = 0.150) (Fig. 6d–f). Of note, brain MAMP was correlated with brain AMP (*R* = 0.945, *p* < 0.001) and OH-MAMP (*R* = 0.619, *p* = 0.014). Day 1 dopamine release did not correlate with serum MAMP (*R* = 0.073, *p* = 0.795) or AMP (*R* = 0.337, *p* = 0.284) (Online Resource 4a,b), and day 1 serotonin release did not correlate with serum MAMP (*R* = 0.030, *p* = 0.917) or AMP (*R* = −0.054, *p* = 0.868) (Online Resource 4c,d). Taken together, this suggests that brain, but not serum, MAMP and AMP concentrations, following MAMP injection on day 1, were responsible for eliciting dopamine and serotonin release in the striatum.

**Fig. 7:**
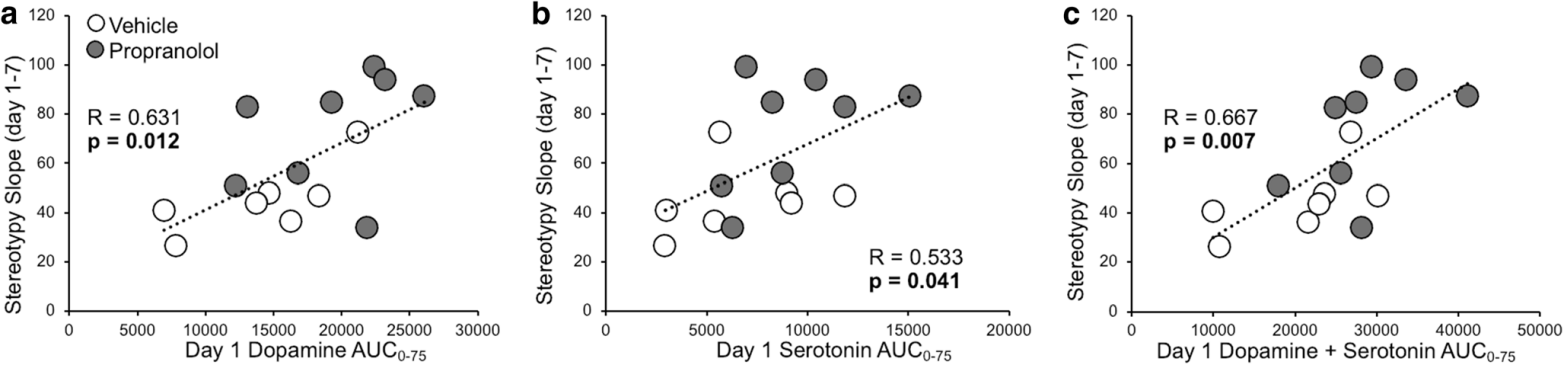
Stereotypy slope correlated with day 1 brain dopamine, serotonin, and the sum of dopamine and serotonin. Rats were given ICV propranolol or vehicle pretreatment 20 h prior to 7 daily MAMP sessions; stereotypy response was recorded daily from 30 to 50 min after injection, and IST microdialysis was conducted on days 1 and 7 (experiment 3). The slope of stereotypy time across sessions correlated with day 1 (**a**) dopamine and (**b**) serotonin AUC_0-75_ and with (**c**) the sum of dopamine and serotonin AUC_0-75_. Correlations were assessed with pretreatment groups combined (*n* = 8 propranolol, 7 vehicle). SD, standard deviation

To examine how neurotransmitter release influenced stereotypy sensitization, relationships between day 1 dopamine and serotonin AUC_0-75_, and the slope of stereotypy time across sessions, were assessed (Fig. 7). Stereotypy slope correlated with day 1 dopamine release (*R* = 0.631, *p* = 0.012) and serotonin release (*R* = 0.533, *p* = 0.041), as well as with the sum of day 1 dopamine and serotonin release (*R* = 0.667, *p* = 0.007). Relationships were also examined between stereotypy slope and the change in dopamine and serotonin release from days 1 to 7 (i.e., day 1 AUC_0-75_ subtracted from day 7 AUC_0-75_), which was used to represent neurotransmitter release sensitization (Online Resource 5). Stereotypy slope did not correlate with the change in dopamine release (*R* = 0.466, *p* = 0.109), the change in serotonin release (*R* = −0.354, *p* = 0.235), or with the change in the sum of dopamine and serotonin release (*R* = 0.348, *p* = 0.244). Taken together, this suggests that dopamine and serotonin release on day 1 specifically, rather than a change in release across days, were more strongly related to the increase in stereotypy response across daily MAMP sessions. In fact, day 7 stereotypy time correlated with day 1 dopamine (*R* = 0.603, *p* = 0.017) and serotonin (*R* = 0.530, *p* = 0.042) release (Online Resource 6a,b) but only trended towards correlating with day 7 dopamine (*R* = 0.520, *p* = 0.057) and did not correlate with day 7 serotonin (*R* = 0.321, *p* = 0.263) release (Online Resource 6c,d). This suggests that dopamine and serotonin release on day 1 were stronger predictors of stereotypy response on day 7 than were dopamine and serotonin release on day 7.

## Discussion

Stereotypy response sensitized across seven daily MAMP injections, and stereotypy sensitization was enhanced by propranolol pretreatment, as compared to vehicle pretreatment, both in the presence and absence of microdialysis conducted during the first and last sessions (i.e., days 1 and 7). Propranolol pretreatment increased brain MAMP concentrations and subsequently enhanced neurotransmitter release assessed in the striatum on days 1 and 7. Brain MAMP and AMP concentrations following MAMP injection on day 1 correlated with striatal dopamine and serotonin release, which correlated with the slope of stereotypy time across daily MAMP sessions, as well as with stereotypy response on day 7. There was no effect of propranolol pretreatment on baseline behavior and neurotransmitter concentrations, or on serum drug concentrations, and serum MAMP and AMP did not correlate with dopamine or serotonin release. This suggests propranolol pretreatment had no relevant impact beyond inhibiting CYP2D in the brain. Taken together, this provides evidence that inhibiting CYP2D in the brain is sufficient to enhance MAMP-induced stereotypy sensitization, likely by enhancing striatal dopamine and serotonin release. This also provides evidence that brain MAMP (and AMP) contribute to acute striatal dopamine and serotonin release and that these initial neurotransmitter responses are strong predictors of subsequent stereotypy sensitization.

Brain MAMP was higher in propranolol-pretreated rats in experiment 2, consistent with propranolol pretreatment inhibiting CYP2D-mediated MAMP metabolism in the brain, consequently increasing brain MAMP concentrations. Likewise, in experiment 2, the brain OH-MAMP/MAMP ratio was reduced in propranolol-pretreated rats, indicating that CYP2D-mediated MAMP *p*-hydroxylation in the brain was inhibited. Conversely, brain AMP and the brain AMP/MAMP ratio were unaffected by propranolol pretreatment. While MAMP *p*-hydroxylation to OH-MAMP is catalyzed entirely by CYP2D, N-demethylation to AMP is catalyzed by CYP2D and other drug metabolizing enzymes in rats (Lin et al. 1995). AMP also readily crosses the blood brain barrier (Melega et al. 1995). Thus, the lack of change in AMP and the AMP/MAMP ratio may be due to AMP being produced from MAMP in the brain by other enzymes, which are unaffected by propranolol, as well as crossing into the brain from the periphery.

CYP2D inhibitor pretreatment enhanced striatal dopamine and serotonin release and behavioral sensitization, indicating that brain MAMP concentrations were increased sufficiently to enhance these responses. One limitation of this study is the high degree of variability in the brain analyte concentrations, which may have reduced our ability to detect a significant increase in brain MAMP concentrations in experiment 3, though we did observe the resulting impact on enhancing neurotransmitter release. Additionally, despite the variability in infrared beam stereotypy measurement in experiment 1, the exacerbation of MAMP-induced stereotypy sensitization by propranolol pretreatment was sufficiently robust to be detected in experiment 1, and this was replicated in experiment 3. MAMP and AMP are approximately equipotent in eliciting behavioral response and sensitization, but under certain conditions, MAMP shows higher potency (da-Rosa et al. 2012; Hall et al. 2008). Thus, both compounds may contribute to behavioral sensitization, but here an increase in brain MAMP concentrations was likely responsible for the enhanced stereotypy sensitization in propranolol-pretreated rats. OH-MAMP and OH-AMP have been proposed to contribute to behavioral sensitization, due to their potential for accumulation in the brain and their ability to elicit behavioral responses via dopaminergic and serotonergic mechanisms (Cho et al. 1975; Dougan et al. 1986; Onogi et al. 2010). However, OH-MAMP and OH-AMP were not detected in brain dialysate collected prior to MAMP injection on day 7, suggesting that these metabolites did not accumulate substantially in the brain under these treatment conditions. Moreover, brain OH-MAMP did not correlate with dopamine or serotonin release, suggesting that it did not contribute in vivo to neurotransmitter release in the dorsal striatum in this study.

Dopamine and serotonin release, assessed on days 1 and 7, were enhanced in rats given propranolol pretreatment, and day 1 MAMP and AMP concentrations correlated with striatal dopamine and serotonin release. In one study, MAMP and AMP (2 mg/kg SC) elicited similar dopamine release in the dorsal striatum, but MAMP was more potent in eliciting striatal serotonin release, with serotonin peak and AUC values 2 to 3 times greater after MAMP, compared to AMP, injection (Kuczenski et al. 1995). Taken together, this suggests some contribution of both MAMP and AMP to dopamine and serotonin release in the dorsal striatum, with greater contribution of MAMP to serotonin release specifically, and that the elevated brain MAMP concentrations in propranolol-pretreated rats were likely responsible for eliciting greater striatal dopamine and serotonin release compared to vehicle-pretreated rats.

Day 1 striatal dopamine and serotonin release correlated with the slope of stereotypy response across sessions, suggesting that dopamine and serotonin signaling in the dorsal striatum may contribute to stereotypy sensitization. Dopamine and serotonin signaling are critical for the acquisition and expression of behavioral sensitization (Ago et al. 2006; Doly et al. 2009; Kuribara and Uchihashi 1994), and signaling in the dorsal striatum specifically is more strongly associated with stereotypy than with other behavioral responses (Sharp et al. 1987). Thus, MAMP-induced dopamine and serotonin release in the dorsal striatum were likely important mediators of stereotypy sensitization. Moreover, the sum of dopamine and serotonin were more strongly correlated with stereotypy slope than were dopamine or serotonin alone, suggesting that the interaction between dopamine and serotonin in the dorsal striatum may contribute meaningfully to MAMP-induced stereotypy sensitization. Thus, in the current study, the increase in MAMP-induced striatal dopamine and serotonin release in propranolol-pretreated rats likely contributed to the exacerbation of subsequent stereotypy sensitization.

Dopamine and serotonin release have been shown to sensitize in several brain regions in parallel with behavioral sensitization (Ago et al. 2006; Kazahaya et al. 1989; Parsons and Justice 1993); however, neurotransmitter release sensitization can occur in the absence of behavioral sensitization and vice versa, suggesting it is not necessary or sufficient for behavioral sensitization to occur (Hamamura et al. 1991; Kuczenski et al. 1997; Shimada et al. 1996). Dopamine, but not serotonin, release trended towards increasing across sessions, suggesting that dopamine release sensitized in the dorsal striatum in parallel with stereotypy sensitization. While the change in dopamine and serotonin release from days 1 to 7 did not correlate with stereotypy slope, dopamine and serotonin release on day 1 did correlate with stereotypy slope, as well as with day 7 stereotypy response. These data suggest a role for acute (i.e., day 1) MAMP-induced striatal neurotransmitter release in subsequent stereotypy response and sensitization across daily MAMP sessions. Acute neurotransmitter release and behavioral response can predict the occurrence and magnitude of behavioral sensitization in some instances (Camp et al. 1994; Kamens et al. 2005; Sills and Vaccarino 1994). Moreover, receptor inhibition and gene knockout studies have implicated dopamine and serotonin signaling in mediating some of the acute drug effects that facilitate subsequent behavioral sensitization (Doly et al. 2009; Valjent et al. 2010; Yoshida et al. 1995). Taken together, this suggests that greater acute MAMP-induced striatal dopamine and serotonin release contributed to the subsequent enhancement of stereotypy sensitization in propranolol-pretreated rats.

Many drugs of abuse, despite having different pharmacological mechanisms, are capable of eliciting behavioral sensitization (Cadoni et al. 2001; Domino 2001; Hall et al. 2008). Behavioral sensitization is associated with addiction and has been used as a model of drug craving and relapse (Robinson and Berridge 1993; Steketee and Kalivas 2011). Animals that exhibit drug-induced behavioral sensitization will often subsequently self-administer more of the drug (Abrahao et al. 2013). Expression of behavioral sensitization is also associated with reinstatement of drug self-administration in animal models (De Vries et al. 1998). The current study provides evidence that CYP2D in the brain can influence brain MAMP concentrations, dopamine and serotonin release in the dorsal striatum, and MAMP-induced behavioral sensitization. Thus, among humans, who exhibit highly variable CYP2D6 in the brain, this impact on brain MAMP concentrations and neurotransmitter release may contribute to individual differences in MAMP response, experience of drug craving and relapse, and susceptibility to other chronic effects of MAMP. To date, most studies in humans have examined the impact of *CYP2D6* genotype on MAMP metabolism and response; one study found that genetically poor (versus extensive) metabolizers were more sensitive to the subjective effects of MAMP, despite there being no difference in serum MAMP AUC, suggesting a possible impact of CYP2D6 in the brain on local drug levels and subsequent response (Sellers and Tyndale 2000). The current study also demonstrated a relationship between acute (i.e., day 1) neurotransmitter release and subsequent stereotypy response and sensitization. This suggests a potentially important influence of initial CNS drug effects, such as MAMP-induced neurotransmitter release, in predicting, and possibly contributing to, subsequent MAMP response and chronic effects. This again highlights the importance of understanding factors, such as CYP2D-mediated MAMP metabolism in the brain, which can influence brain drug concentrations and resulting neurotransmitter release and behavioral responses.

## Supplementary information

Online Resource 1 (PDF 33 kb)

Online Resource 2 (PDF 33 kb)

Online Resource 3 (PDF 39 kb)

Online Resource 4 (PDF 38 kb)

Online Resource 5 (PDF 35 kb)

Online Resource 6 (PDF 48 kb)
