# Supplementary material for: The role of CYP2D in rat brain in methamphetamine-induced striatal dopamine and serotonin release and behavioral sensitization
Source: Psychopharmacology (Berl). 2021 Mar 1;238(7):1791–804. doi: 10.1007/s00213-021-05808-9 (PMC8233297; doi:10.1007/s00213-021-05808-9)

Psychopharmacology

The role of CYP2D in rat brain in methamphetamine-induced striatal dopamine and serotonin release and behavioral sensitization

Marlaina R Stocco, Ahmed A El-Sherbeni, Bin Zhao, Maria Novalen, Rachel F Tyndale

Corresponding author: Dr. Rachel F Tyndale
Departments of Pharmacology & Toxicology, Psychiatry, University of Toronto
Email address: r.tyndale@utoronto.ca

Online Resource 1 Propranolol (versus vehicle) pretreatment had no effect on serum drug concentrations. Rats were given propranolol (n = 3 ICV, 3 IST) or vehicle (n = 3 ICV, 3 IST) pretreatment 20 hr prior to a single MAMP injection, and blood samples were taken at 100 and 130 min after injection (Experiment 2). Serum (**a**) MAMP and (**b**) AMP, and the (**c**) AMP/MAMP ratio, did not differ between pretreatments. OH-MAMP concentrations in serum were below the LOQ in most samples and are not reported here. Veh, vehicle; Prl, propranolol; SD, standard deviation.

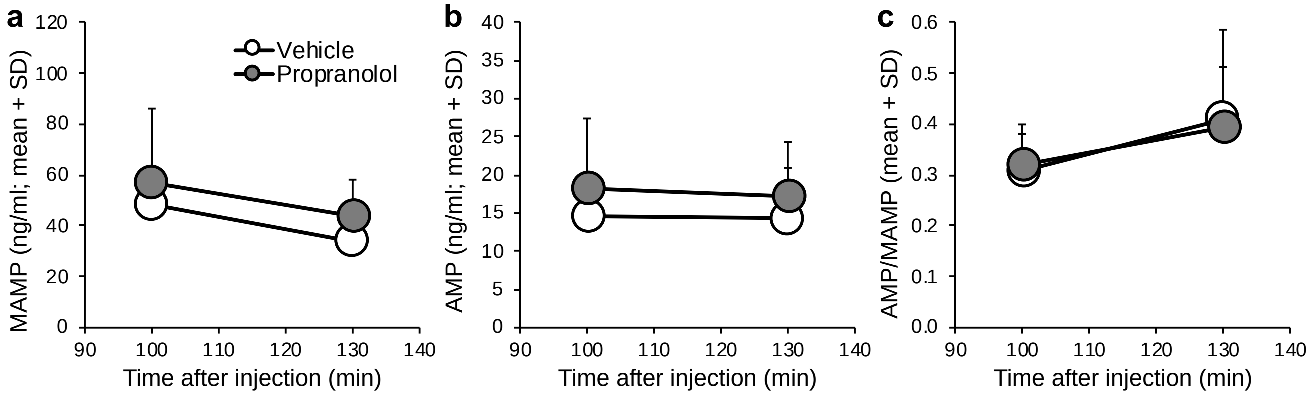

Supplement: Supplementary file 1 — (PDF 33 kb) [file 213_2021_5808_MOESM1_ESM.pdf]
