# Supplementary material for: The role of CYP2D in rat brain in methamphetamine-induced striatal dopamine and serotonin release and behavioral sensitization
Source: Psychopharmacology (Berl). 2021 Mar 1;238(7):1791–804. doi: 10.1007/s00213-021-05808-9 (PMC8233297; doi:10.1007/s00213-021-05808-9)

Psychopharmacology

The role of CYP2D in rat brain in methamphetamine-induced striatal dopamine and serotonin release and behavioral sensitization

Marlaina R Stocco, Ahmed A El-Sherbeni, Bin Zhao, Maria Novalen, Rachel F Tyndale

Corresponding author: Dr. Rachel F Tyndale
Departments of Pharmacology & Toxicology, Psychiatry, University of Toronto
Email address: r.tyndale@utoronto.ca

Online Resource 2 ICV propranolol (versus vehicle) pretreatment enhanced the repeated MAMP-induced decrease in rearing response. Rats were given ICV propranolol (n = 8) or vehicle (n = 8) pretreatment 20 hr prior to 7 daily MAMP sessions, and rearing response was recorded daily from 15-45 min after injection (Experiment 3). Day 1 rearing events were excluded due to rearing behavior being disrupted by the microdialysis apparatus on day 1. (**a**) Total rearing events decreased across MAMP sessions (day 2-7) in propranolol-pretreated rats. (**b**) The slope of rearing events across sessions trended towards being lower in propranolol-pretreated rats. Day versus day 2: #p < 0.05, ##p < 0.01, ###p < 0.001. Veh, vehicle; Prl, propranolol; SD, standard deviation.

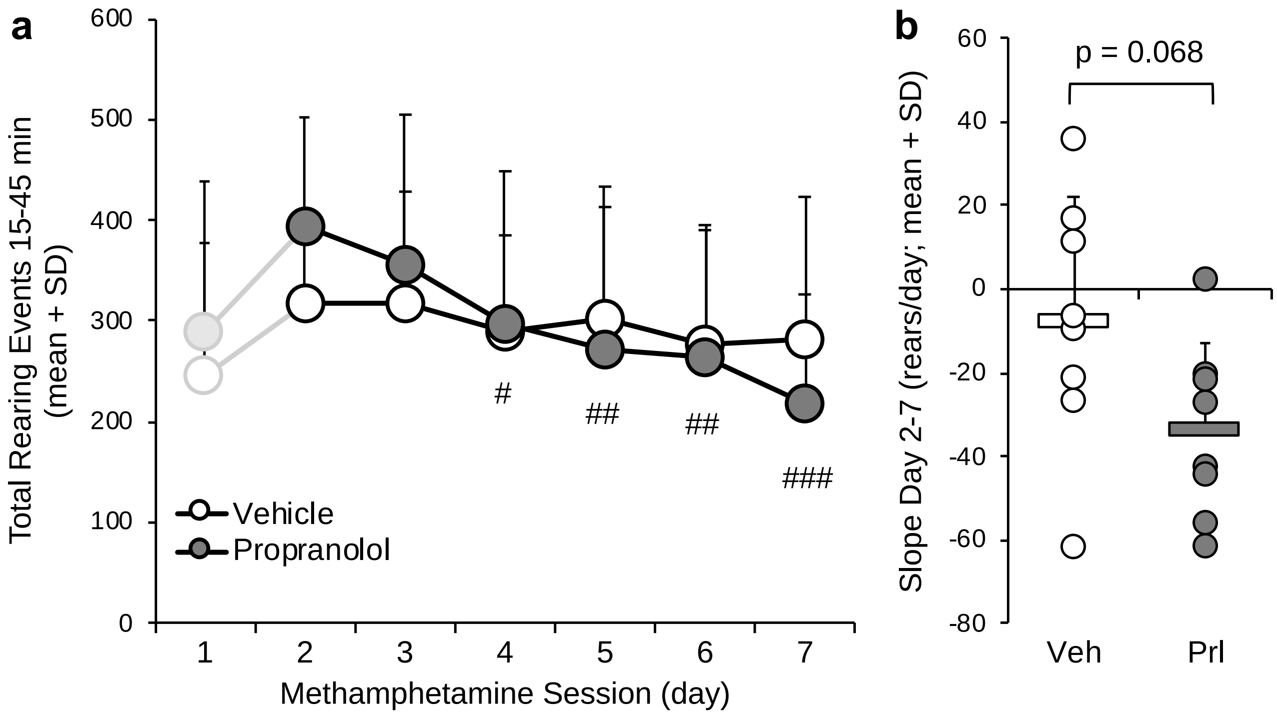

Supplement: Supplementary file 2 — (PDF 33 kb) [file 213_2021_5808_MOESM2_ESM.pdf]
