# Supplementary material for: The role of CYP2D in rat brain in methamphetamine-induced striatal dopamine and serotonin release and behavioral sensitization
Source: Psychopharmacology (Berl). 2021 Mar 1;238(7):1791–804. doi: 10.1007/s00213-021-05808-9 (PMC8233297; doi:10.1007/s00213-021-05808-9)

Psychopharmacology

The role of CYP2D in rat brain in methamphetamine-induced striatal dopamine and serotonin release and behavioral sensitization

Marlaina R Stocco, Ahmed A El-Sherbeni, Bin Zhao, Maria Novalen, Rachel F Tyndale

Corresponding author: Dr. Rachel F Tyndale
Departments of Pharmacology & Toxicology, Psychiatry, University of Toronto
Email address: r.tyndale@utoronto.ca

Online Resource 3 ICV propranolol (versus vehicle) pretreatment had no effect on serum drug concentrations. Rats were given ICV propranolol (n = 8) or vehicle (n = 8) pretreatment 20 hr prior to 7 daily MAMP sessions, and blood samples were taken at 100 and 130 min after injection on day 1 (top) and day 7 (bottom) (Experiment 3). Serum (**a,d**) MAMP, (**b,e**) AMP, and the (**c,f**) AMP/MAMP ratio did not differ between pretreatments. OH-MAMP concentrations were below the LOQ in most samples and are not reported here; day 1 AMP concentrations were below the LOQ in three samples, and data graphed in (**b,c**) are from the remaining animals (n = 6 propranolol, 7 vehicle). Veh, vehicle; Prl, propranolol; SD, standard deviation.

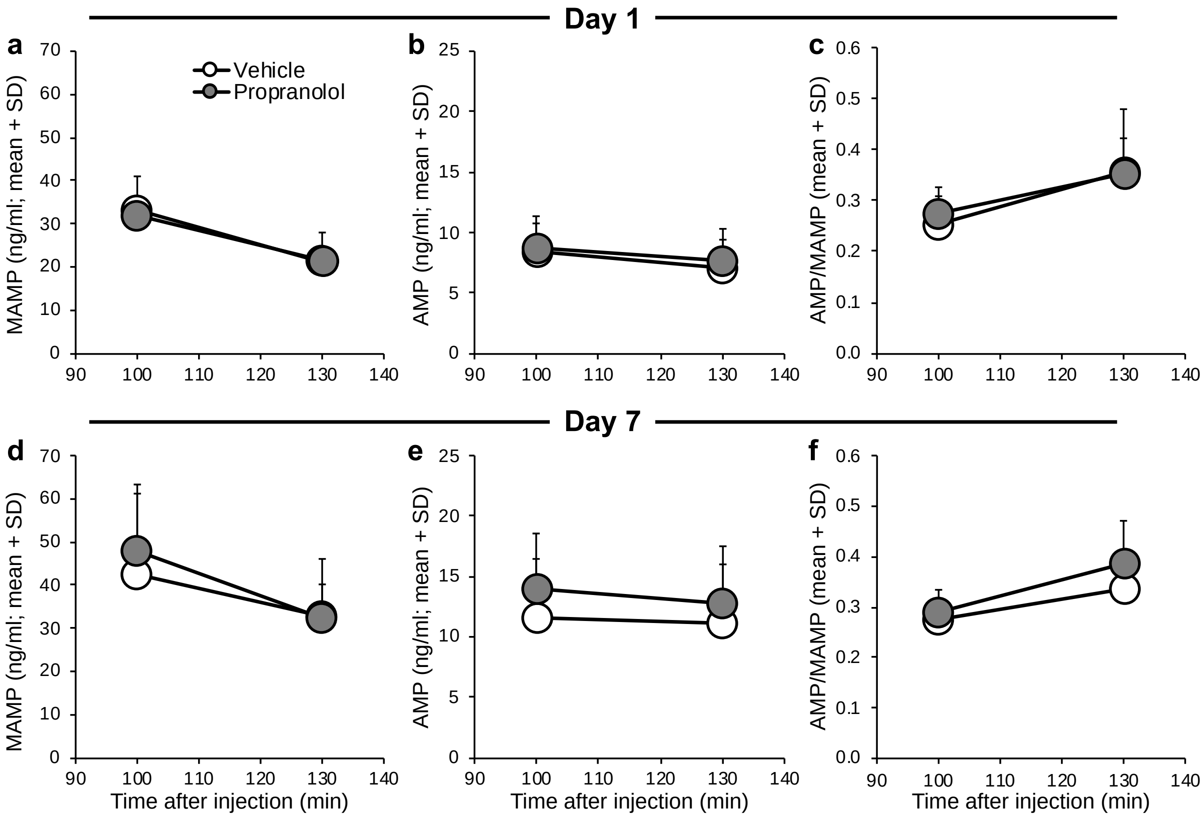

Supplement: Supplementary file 3 — (PDF 39 kb) [file 213_2021_5808_MOESM3_ESM.pdf]
