# Supplementary material for: The role of CYP2D in rat brain in methamphetamine-induced striatal dopamine and serotonin release and behavioral sensitization
Source: Psychopharmacology (Berl). 2021 Mar 1;238(7):1791–804. doi: 10.1007/s00213-021-05808-9 (PMC8233297; doi:10.1007/s00213-021-05808-9)

Psychopharmacology

The role of CYP2D in rat brain in methamphetamine-induced striatal dopamine and serotonin release and behavioral sensitization

Marlaina R Stocco, Ahmed A El-Sherbeni, Bin Zhao, Maria Novalen, Rachel F Tyndale

Corresponding author: Dr. Rachel F Tyndale
Departments of Pharmacology & Toxicology, Psychiatry, University of Toronto
Email address: r.tyndale@utoronto.ca

Online Resource 4 Day 1 dopamine and serotonin did not correlate with serum MAMP and AMP. Rats were given ICV propranolol or vehicle pretreatment 20 hr prior to 7 daily MAMP sessions; IST microdialysis was conducted, and blood samples were collected on day 1 and day 7 (Experiment 3). Day 1 dopamine AUC_{0-75} did not correlated with day 1 serum (**a**) MAMP or (**b**) AMP concentrations assessed at 100 min. Day 1 serotonin AUC_{0-75} did not correlated with serum (**c**) MAMP or (**d**) AMP concentrations assessed at 100 min. Correlations were assessed with pretreatment groups combined, and AMP concentrations below the LOQ were excluded (MAMP data in (**a,c**): n = 8 propranolol, 7 vehicle; AMP data in (**b,d**): n = 6 propranolol, 6 vehicle).

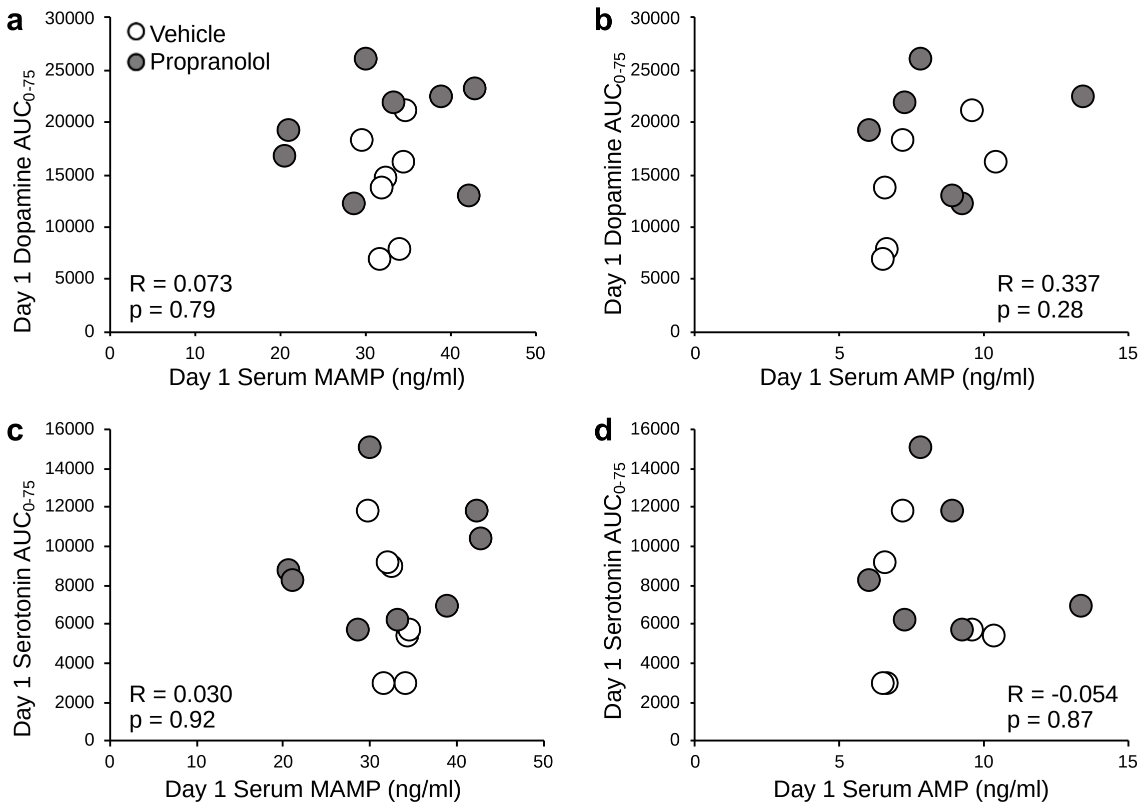

Supplement: Supplementary file 4 — (PDF 38 kb) [file 213_2021_5808_MOESM4_ESM.pdf]
