# Supplementary material for: The role of CYP2D in rat brain in methamphetamine-induced striatal dopamine and serotonin release and behavioral sensitization
Source: Psychopharmacology (Berl). 2021 Mar 1;238(7):1791–804. doi: 10.1007/s00213-021-05808-9 (PMC8233297; doi:10.1007/s00213-021-05808-9)

Psychopharmacology

The role of CYP2D in rat brain in methamphetamine-induced striatal dopamine and serotonin release and behavioral sensitization

Marlaina R Stocco, Ahmed A El-Sherbeni, Bin Zhao, Maria Novalen, Rachel F Tyndale

Corresponding author: Dr. Rachel F Tyndale
Departments of Pharmacology & Toxicology, Psychiatry, University of Toronto
Email address: r.tyndale@utoronto.ca

Online Resource 6 Stereotypy time on day 7 correlated with day 1, but not day 7, dopamine and serotonin. Rats were given ICV propranolol or vehicle pretreatment 20 hr prior to 7 daily MAMP sessions; stereotypy response was recorded daily from 30-50 min after injection, and IST microdialysis was conducted on day 1 and on day 7 (Experiment 3). Total stereotypy time on day 7 correlated with day 1 (**a**) dopamine and (**b**) serotonin AUC_{0-75}, but it did not correlate with day 7 (**c**) dopamine or (**d**) serotonin AUC_{0-75}. Correlations were assessed with pretreatment groups combined (day 1 data in (**a,b**): n = 8 propranolol, 7 vehicle; day 7 data in (**c,d**): n = 6 propranolol, 8 vehicle).

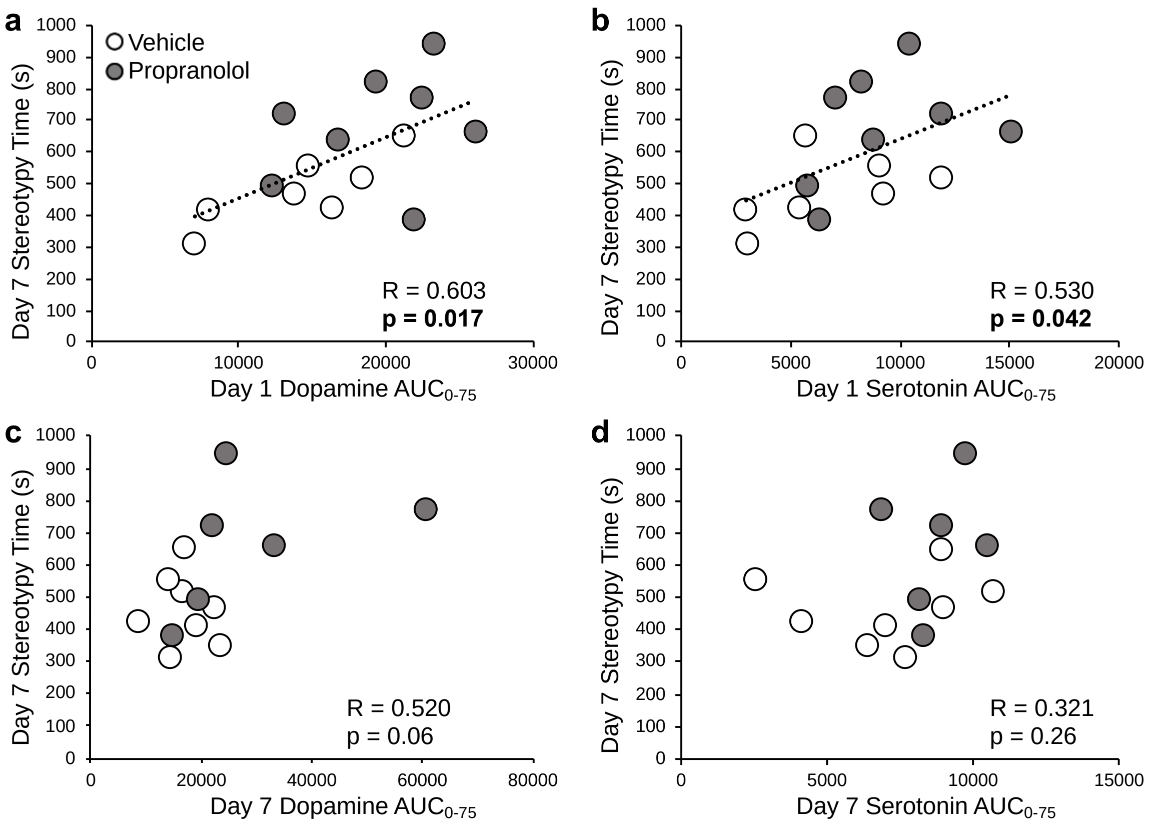

Supplement: Supplementary file 6 — (PDF 48 kb) [file 213_2021_5808_MOESM6_ESM.pdf]
